# Molecular Recognition of Methacryllysine and Crotonyllysine by the AF9 YEATS Domain

**DOI:** 10.3390/ijms24087002

**Published:** 2023-04-10

**Authors:** Nurgül Bilgin, Laust Moesgaard, Mohammad M. Rahman, Vildan A. Türkmen, Jacob Kongsted, Jasmin Mecinović

**Affiliations:** Department of Physics, Chemistry and Pharmacy, University of Southern Denmark, Campusvej 55, 5230 Odense, Denmark; bilgin@sdu.dk (N.B.); moesgaard@sdu.dk (L.M.); mubinur.rahman@oulu.fi (M.M.R.); vildan@sdu.dk (V.A.T.)

**Keywords:** crotonylation, epigenetics, histone, methacrylation, molecular recognition, posttranslational modifications, YEATS domain

## Abstract

Histone lysine methacrylation and crotonylation are epigenetic marks that play important roles in human gene regulation. Here, we explore the molecular recognition of histone H3 peptides possessing methacryllysine and crotonyllysine at positions 18 and 9 (H3K18 and H3K9) by the AF9 YEATS domain. Our binding studies demonstrate that the AF9 YEATS domain displays a higher binding affinity for histones possessing crotonyllysine than the isomeric methacryllysine, indicating that AF9 YEATS distinguishes between the two regioisomers. Molecular dynamics simulations reveal that the crotonyllysine/methacryllysine-mediated desolvation of the AF9 YEATS domain provides an important contribution to the recognition of both epigenetic marks. These results provide important knowledge for the development of AF9 YEATS inhibitors, an area of biomedical interest.

## 1. Introduction

**Figure 1:**
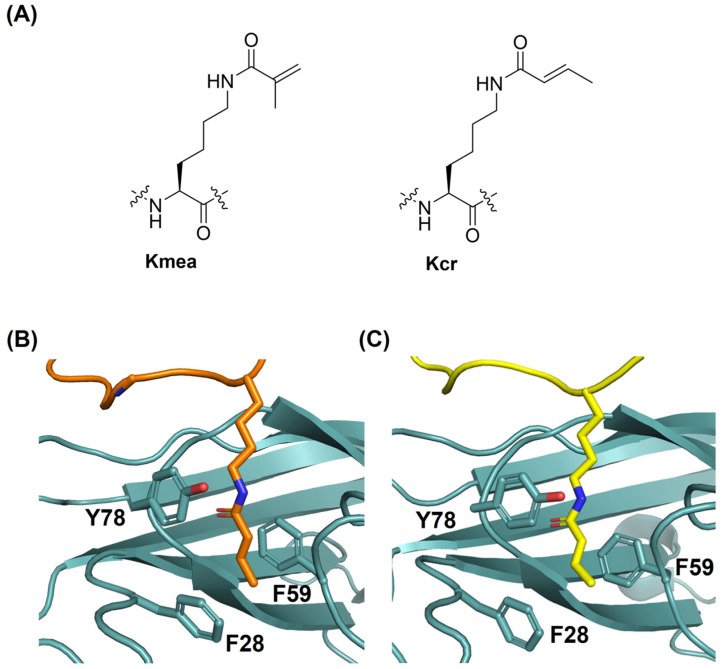
Recognition of methacryllysine and crotonyllysine by the AF9 YEATS domain. (**A**) Structures of methacryllsyine (Kmea) and crotonyllysine (Kcr). (**B**) A view of the crystal structure of AF9 YEATS (cyan) complexed with histone H3K18cr (orange) (PDB ID: 2NDG). (**C**) A view of the crystal structure of AF9 YEATS (cyan) complexed with histone H3K9cr (yellow) (PDB ID: 5HJB).

Histone proteins are subject to diverse posttranslational modifications (PTMs), also known as epigenetic marks [1,2]. Histone lysine PTMs, such as methylation and acetylation, have crucial roles in cellular processes in eukaryotes, including DNA replication, DNA repair, and regulation of gene expression [1,2,3]. Various types of histone marks can be derived from acyl-CoA metabolites [4]. These comprise lysine acetylation (Kac) and related lysine acylations, including formylation (Kfo), propionylation (Kpr), butyrylation (Kbu), benzoylation (Kbz), crotonylation (Kcr), and methacrylation (Kmea) [4,5,6,7,8]. Acyl-CoA acts as a cosubstrate in acyltransferase-catalysed lysine acylations [4]. Histone lysine crotonylation (Kcr), an enzymatically regulated epigenetic modification, has diverse biological roles related to numerous disease states (Figure 1A) [4,9,10]. CBP/p300 enzymes are the major histone crotonyltranferases that introduce the crotonyl moiety on lysine, resulting in stimulation of transcription to a higher degree than histone acetylation [9,10,11]. Very recently, lysine methacrylation (Kmea) was discovered as a new type of histone PTM (Figure 1A) [8]. This modification was identified on 27 different histone sites in HeLa cells and verified by specific anti-methacryllysine antibodies and mass spectrometric approaches [8]. Biochemical studies showed that Kmea is a dynamic mark that is controlled by HAT1 as a methacryltransferase and SIRT2 as a demethacrylase [8]. In addition, the methacrylyl-CoA-generating metabolism suggests that the isomeric Kcr and Kmea could be connected to different regulatory pathways [8].

Histone PTMs are recognised by chromatin-binding protein modules, also known as reader domains, which mediate downstream biological processes [12,13]. Three major acylation reader families have been identified in humans, including bromodomain (BRD) [14], double PHD finger (DPF) domain [15], and YEATS (Yaf9, ENL, AF9, Taf14, and Sas5) domain proteins [16]. BRD and DPF domains were characterised as Kac readers that bind Kac through a hydrophobic pocket via hydrogen bonding. The YEATS domain possesses an aromatic sandwich pocket for the recognition of acyllysine residues [17,18]. The recognition pockets of the YEATS and DPF domains favour the binding of Kcr over Kac, in contrast to BRD, which is a Kac-specific domain [17]. YEATS domain proteins represent a key family of reader proteins that preferentially recognise Kcr on histones over other acyl modifications, while the AF9 YEATS directly links the recognition of Kcr to active transcription [16,19]. The YEATS domain of AF9 is known to recognise acyllysine residues at multiple positions on histone H3, including K9, K18, and K27 [17,18]. Molecular evidence suggests that the planar crotonyl group outcompetes other lysine acylations found in nature in that it contains an α,β-unsaturated short hydrocarbon chain [19]. This molecular feature mediates the π-π-π stacking interactions, where the amide and alkene moieties of Kcr are bound between two highly conserved aromatic side chains of AF9 YEATS (Tyr78 and Phe59, Figure 1B,C) [17,19]. Tyr78 is primarily responsible for the amide group recognition through amide-π and hydrogen bond interactions, while Phe59 provides critical alkene-π stacking contacts with the crotonyl hydrocarbon chain [17,19,20]. The terminal methyl group of Kcr is apparently recognised by a CH-π mediated contact with the Phe28 residue of AF9 YEATS (Figure 1B,C) [17,21]. Due to its biomedical importance, peptidomimetic and small molecule inhibitors of AF9 YEATS have been developed by targeting the π-π-π stacking interaction at the Kcr recognition site [22,23,24].

Histone lysine PTMs are dynamically regulated by epigenetic writers, erasers, and readers, as is firmly established for methylation, acetylation, and crotonylation [6,10,25]. While the newly discovered Kmea has been examined for its formation by acetyltransferase enzymes (i.e., writers) and removal by deacetylase enzymes (i.e., erasers), it is currently unknown whether the YEATS domain has an affinity for Kmea and therefore acts as a reader of Kmea [8]. In this study, we aim to comparatively examine the molecular recognition of Kmea and the regioisomeric Kcr by the AF9 YEATS domain using binding analyses and molecular dynamics simulations.

## 2. Results and Discussion

To examine the molecular readout of Kmea and Kcr by the AF9 YEATS domain, we synthesised methacrylated and crotonylated histone peptides H3K9 (residues 3–15, sequence: TKQTARKSTGGKA) and H3K18 (residues 10–25, sequence: STGGKAPRKQLATKAA). All histone H3 peptides were prepared on resin by Fmoc-based solid-phase peptide synthesis (SPPS) employing an orthogonal alloc group on K9 or K18, respectively. H3K9 and H3K18 peptides were selectively acylated by performing on-resin reactions with methacryl chloride or crotonyl chloride in the presence of triethylamine (Figure S1). Mass spectrometry and analytical HPLC confirmed the purity of all synthetic histone peptides (Figures S2–S11 and Table S1). The AF9 YEATS domain was chosen because it has been structurally characterised and binds H3K18cr and possibly H3K18mea, two naturally occurring epigenetic marks [8]. The recombinant human AF9 YEATS domain (residues 1–138) was expressed in *E. coli* and purified by Ni-column and size exclusion chromatography (Figure S12).

**Figure 2:**
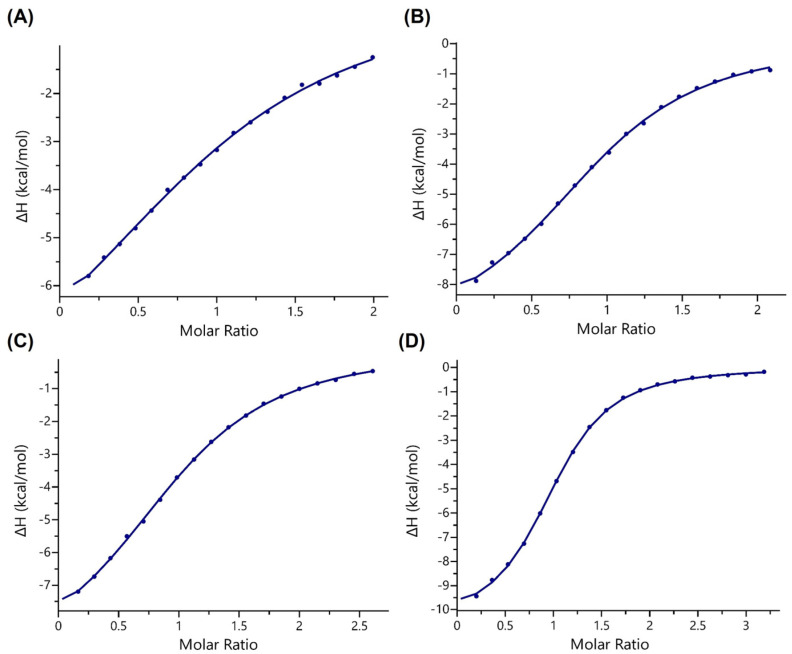
Representative ITC binding curves of AF9 YEATS titrated by methacrylated and crotonylated H3K18 and H3K9 peptides. (**A**) H3K18mea, (**B**) H3K18cr, (**C**) H3K9mea, and (**D**) H3K9cr.

Given the novel discovery of the methacryl mark on the H3K18 site, isothermal titration calorimetry (ITC) experiments were first carried out with synthetic H3K18mea and H3K18cr peptides (Figure 2 and Figures S13 and S14). The binding analyses reveal that the human AF9 YEATS domain displays weaker binding affinity for H3K18mea (K_d_ = 114 μM) than for H3K18Kcr (K_d_ = 21 μM), suggesting that AF9 YEATS discriminates between the regioisomeric Kmea and Kcr (Table 1, Figure 2). Having shown the preferential binding of Kcr over Kmea at the H3K18 site, we continued our investigations by performing ITC experiments with modified H3 peptides at the K9 site to compare the binding preference between H3K9mea and H3K9cr. AF9 YEATS displays ~6-fold reduced affinity for H3K9mea relative to H3K9cr (K_d_ = 59 μM H3K9mea vs. K_d_ = 11 μM H3K9cr), indicating the superior binding affinity for Kcr over Kmea regardless of the acylation site (Table 1). Differences in the binding free energy of methacrylated and crotonylated histones are small but noticeable (ΔΔG ~1.0 kcal mol^−1^). These findings indicate that the binding mechanisms between both regioisomeric epigenetic marks might be different and raise the question of whether key noncovalent interactions between AF9 YEATS and Kmea/Kcr are conserved in the AF9 YEATS-H3 complex, and whether desolvation of the Kmea/Kcr recognition site of AF9 YEATS contributes to differences in the molecular readout of both epigenetic marks.

**Figure 3:**
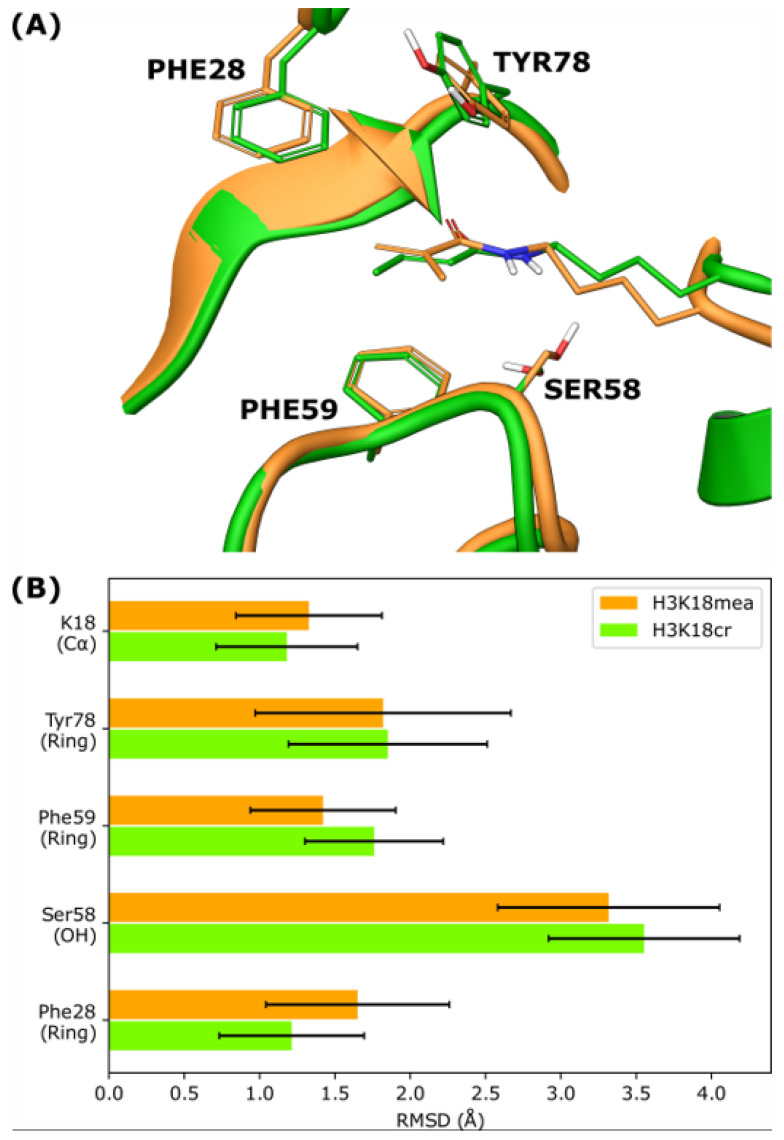
Molecular dynamics simulations. (**A**) Average structures of the AF9 YEATS methacryllysine/crotonyllysine binding site from the 500 ns MD simulations of H3K18mea (orange) and H3K18cr (green). (**B**) Root mean square deviations of selected atom groups compared to the starting H3K18cr structure.

To predict and compare methacryllysine and crotonylllysine binding to the AF9 YEATS domain, molecular dynamics (MD) simulations of the AF9 YEATS domain in complex with H3K18mea and H3K18cr peptides were performed. These simulations showed very similar binding conformations for both peptides compared to previously determined structures of H3K18cr and H3K9cr bound to AF9 YEATS (Figure 3A and Figure S15) [19]. However, when considering the correlated movements of the side chain heavy atoms K18mea and K18cr, a significantly larger correlation was observed for H3K18cr (Figure S16). This indicates a more stable side chain conformation for H3K18cr compared to H3K18mea (Figure S16A,B). Specifically, the correlation coefficients for H3K18cr show three regions with high correlations separated by the dihedrals defined by the C_β_-C_γ_ and C_δ_-C_ε_ bonds of Kcr/Kmea. This finding suggests that these dihedrals are responsible for the stabilisation and optimal positioning of the remainder of the side chain. In general, the conformation of the Kmea/Kcr binding site in the simulations drifts only negligibly from the starting AF9 YEATS conformation, and very similar effects were observed for both H3K18cr and H3K18mea simulations (Figure 3B and Figure S17). However, significant movements are observed for Ser58, which was found to move away from the lysine residue in both simulations, thus preventing hydrogen bonding to the acyl amide NH (Figure S18). Conversely, relatively stable hydrogen bonding between the amide carbonyl of Kmea/Kcr and the backbone of Tyr78 was observed (Figure S19), indicating that this interaction is highly important for stabilising the binding of both peptides. During the simulations, both Kmea and Kcr remained in contact with Phe28 (Figure S20A), further supporting the existence of weak CH-π interactions [21]. For Kmea, however, the contact was through the methylidene group instead of the methyl group that forms the contact for Kcr (Figure S20B).

**Figure 4:**
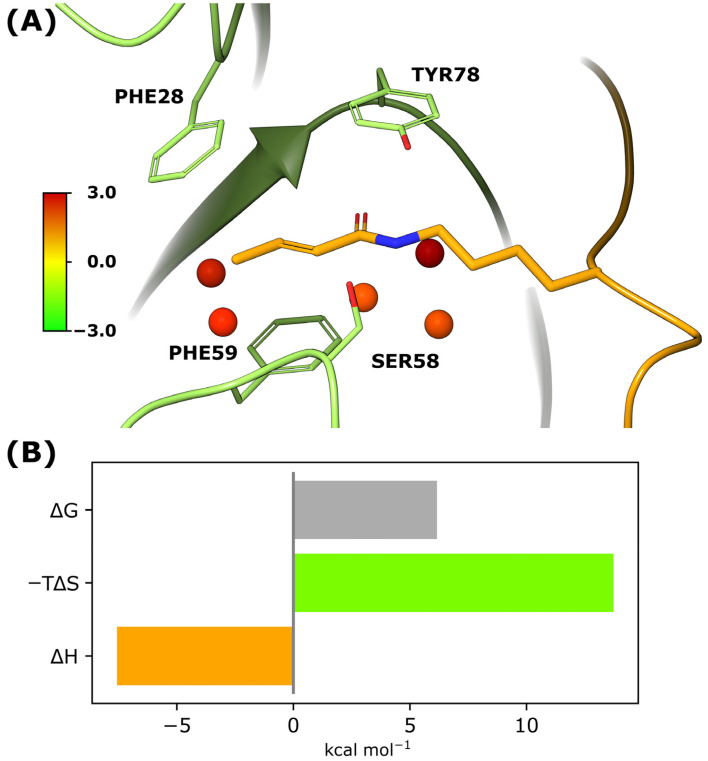
Thermodynamics of water molecules inside the aromatic pocket of the AF9 YEATS domain. (**A**) Identified hydration sites at the methacryllysine/crotonyllysine binding pocket of AF9 YEATS, determined by GIST. (**B**) Approximated solvation energy terms at the methacryllysine/crotonyllysine binding pocket of AF9 YEATS.

As the Kmea/Kcr binding site forms a relatively hydrophobic valley in the AF9-H3 complex, we hypothesised that the Kmea/Kcr-mediated desolvation of the binding site contributes significantly to the favourable binding of methacryllysine and crotonyllysine. To investigate the thermodynamics of water inside the Kmea/Kcr binding site, Grid Inhomogeneous Solvation Theory (GIST) was used to estimate the energetic terms associated with the solvation of the binding site [26,27]. By applying GIST to a simulation of the unbound AF9 YEATS domain, four relatively high-energy hydration sites were found to occupy the Kmea/Kcr binding site (Figure 4A and Figure S21). This observation suggests that through the binding of Kmea/Kcr, water molecules that occupy the aromatic pocket are released, which consequently lowers the energy of the system. In total, the free energy of the Kmea/Kcr binding site solvation was estimated to be 6.0 kcal mol^−1^, which is primarily due to the entropic term (13.6 kcal mol^−1^), associated with the restricted rotation/translation of water molecules inside the binding pocket (Figure 4B). Interestingly, the enthalpic contribution to the solvation energy was estimated to be favourable (−7.6 kcal mol^−1^), as broken water-water interactions are more than fully compensated by water-solute interactions. It was estimated that water-water interactions and water-solute interactions contribute almost equally to the solvation energy (−36.6 kcal mol^−1^ vs. −33.0 kcal mol^−1^) for water molecules inside the Kmea/Kcr binding site.

When comparing the binding of H3K18mea and H3K18cr, there is a noticeable difference in the desolvation energy of the areas where water is repelled by the methyl group of H3K18cr relative to the methyl group of H3K18mea (Figure S22). This suggests that H3K18cr binding to AF9 YEATS leads to a higher release of energy due to desolvation than does H3K18mea binding. The volumes of the desolvated water molecules were estimated to be 62 Å^3^ and 80 Å^3^ for H3K18cr and H3K18mea, respectively (Figure S23). These differences in the desolvation energies are primarily caused by differences in the enthalpic term (3.5 kcal mol^−1^ vs. 10.4 kcal mol^−1^), whereas the entropic desolvation term is almost identical for the two (10.7 kcal mol^−1^ vs. 10.9 kcal mol^−1^). Overall, these results suggest that desolvation of the Kmea/Kcr binding site plays an important role in methacryllysine and crotonyllysine recognition by the AF9 YEATS domain.

## 3. Materials and Methods 

### 3.1. Synthesis of Histone Peptides

All histone H3 peptides were manually synthesised employing Fmoc-SPPS chemistry on Rink amide resin (0.78 mmol/g loading capacity) and modified on-resin from alloc-protected K18 or K9 side chains (H3K18alloc_10–25_ or H3K9alloc_3–15_). The coupling of the amino acids (3 eq.) was carried out at room temperature for 1 h upon activation with HATU (2.9 eq.) and DIPEA (6.0 eq.) in DMF. Fmoc deprotection was achieved by swelling with a solution of 20% piperidine in DMF for 20 min. After each coupling and deprotection step, a Kaiser test was done to ensure completion of the reaction. The final N-terminal amino acid was coupled as a Nα-Boc amino acid. Upon completion of the sequence, fully protected peptides with incorporated Lys(Alloc) at positions 18 or 9 were swollen in DCM under nitrogen flow for 10 min at room temperature. The peptide was proceeded with for the orthogonally removal of the alloc protecting group on-resin by treatment with phenylsilane (24 eq.) and tetrakis(triphenylphosphine)-palladium (1.0 eq.) to the N_2_-bubbling resin in DCM, and the reaction continued under nitrogen flow for 1 h at room temperature. Extensive washing of the resin with DCM, DMF, and sodium diethyldithiocarbamate (0.5% in DMF) was followed by a Kaiser test to monitor deprotection. To deprotected resin was added TEA (1.5 eq.) in DCM and agitated for 10 min at room temperature. Subsequently, addition of either cronotyl chloride (3.0 eq.) or methacroloyl chloride (3.0 eq.) in DCM was allowed to proceed for 2 h, and the reaction was monitored by the Kaiser test (Figure S1). The final peptides were washed with methanol, dichloromethane, and dried over diethyl ether, and then proceeded to cleave from resin using 95% TFA, 2.5% TIPS, and 2.5% MQ-water for 4 h. Next, the crude peptides were precipitated with cold diethyl ether (−20 °C) and pelleted via centrifugation. Finally, the synthetic modified histone peptides were purified by RP-HPLC, and their purity was assessed by MALDI-TOF MS and analytical RP-HPLC (Figures S2–S11). 

### 3.2. Expression and Purification of the AF9 YEATS Domain

The plasmid vector pET28b(+) containing the cloned AF9 YEATS gene (amino acids 1–138) with an N-terminal 6xHis tag was obtained as a gift from Prof. Haitao Li (Tsinghua University, China) [17]. The plasmid was transformed into Rosetta 2(DE_3_)pLysS *E. coli* cells (Novagen, Darmstadt, Germany) by the heat-shock method. Transformed cells were cultivated at 37 °C in TB medium supplemented with 50 μg/mL kanamycin and 35 μg/mL chloramphenicol. The cultivation continued until the culture’s OD_600_ reached 1.5. Then the culture was cooled down to room temperature (RT), and the protein expression was induced by adding isopropyl β-d-1-thiogalactopyranoside (IPTG) at a final concentration of 0.3 mM. The cultivation was continued for 18 h at RT. After the protein expression was completed, the cells were harvested by centrifuging the culture at 6500 rpm at 4 °C for 15 min. The pellets were then resuspended in cell lysis buffer containing 50 mM Tris, 500 mM NaCl, 30 mM imidazole, at pH 7.4, 1 mM phenylmethylsulfonyl fluoride (PMSF), 2 mM β-mercaptaethanol (β-ME), and 20 μg/mL DNase I. Subsequently, the cells were lysed using the One Shot Cell Disrupter (Constant Systems LTD, Daventry, UK), and the cell debris was settled down by centrifuging the lysate at 19500 rpm at 4 °C for 20 min. The supernatant was filtered through a 0.2 μm syringe filter. 

Protein purification was carried out using the chromatography system ÄKTA™ pure (Cytiva, Uppsala, Sweden). The crude protein was loaded onto a pre-packed HisTrap Ni-NTA column (Cytiva) equilibrated with a binding buffer: 50 mM Tris, 500 mM NaCl, 30 mM Imidazole, pH 7.4, and 2 mM DTT. The bound 6xHis-tagged AF9 YEATS protein was then eluted with a linear gradient up to 100% using an elution buffer: 50 mM Tris, 500 mM NaCl, 300 mM Imidazole, at pH 7.4, and 2 mM DTT. The fractions were checked by SDS-PAGE, and 6xHis-AF9 YEATS-containing fractions were pooled and concentrated using Vivaspin 20 MWCO 10 kDa (Cytiva). The final polishing of the protein sample was carried out using Superdex 75 10/300, a size exclusion chromatography (SEC) column (Cytiva), equilibrated with SEC buffer: 50 mM sodium phosphate, 500 mM NaCl, 2 mM EDTA at pH 7.4, and 2 mM β-ME. The large peak fractions assumed to be the monomeric 6xHis AF9 YEATS were checked by SDS-PAGE (Figure S12A).

### 3.3. ITC Binding Assays 

ITC experiments were performed using a MicroCal PEAQ-ITC instrument (Malvern Panalytical, Malvern, UK). For the titrations, all synthetic histone peptides (H3K18cr, H3K18mea, H3K9cr, and H3K9mea) and the recombinant AF9 YEATS domain were dissolved in the same ITC buffer (50 mM NaH_2_PO_4_, 500 mM NaCl, 2 mM EDTA, and 2 mM β-ME). The concentrations of all histone peptides and proteins were measured by UV analysis on a Thermo-Fisher Nanodrop spectrophotometer in buffer using λ_max_ at 215 nm and 280 nm, respectively. H3 peptides (1.1–2.7 mM) were titrated into the AF9 YEATS protein at (94–205 μM) with 19 injections, 0.4 μL for the first injection and 2.0 μL for the rest. ITC fitting curves were processed using the One Set of Sites model in the MicroCal ITC analysis software, version 1.40. 

### 3.4. Molecular Dynamics Simulations 

The structure of AF9 YEATS (PDB ID: 2NDG) [19] was acquired from the PDB database and imported into Maestro, which is available with the Schrödinger Suite [28]. Here, the first entry was extracted and exported. Using the Build module in Maestro, the crotonyl residue was changed to methacryloyl and exported again. GAFF parameters for crotonyl and methacryloyl were determined using the AM1-BCC charge method in Antechamber, while the protein was treated with FF19SB parameters [29,30,31,32]. 

Tleap was used to construct parameter files and start structures for the systems. Three systems were constructed: one with AF9 bound to H3K18cr as in the original PDB file, one where crotonyl was changed to methacryl, and one without an H3K18 peptide. Each of the systems was solvated in a cubic TIP3P water box with a NaCl concentration of 0.150 M and counter ions to neutralise them. The minimum distance between the protein and the sides of the applied simulation box was 11 Å. 

All MD simulations were performed using Amber and Particle Mesh Eward (PME), a nonbonded cutoff of 10.0 Å, a time step of 2 fs, and the SHAKE algorithm to treat bonds as hydrogens [33,34,35]. All systems were initially minimised for 500 steps using the steepest descent algorithm, followed by 500 steps using the conjugate gradient algorithm. The minimizations were performed with restraints on the protein backbone. The systems were then annealed from 0 K to 300 K for 50 ps in the NVT ensemble using the Langevin thermostat while maintaining the backbone restrains [36]. After this, the Berendsen barostat was applied to control the pressure. After 50 ps of simulation in the NPT ensemble, the restraints were lifted, and 10 ns of simulation with production settings was performed to equilibrate the systems further. The systems were then simulated for 500 ns while sampling.

To investigate the water dynamics of the crotonyl binding site, the endpoint of the apo simulation was used as a starting point for a new 100 ns MD simulation in the NVT ensemble with restraints on the protein. Snapshots were extracted every 1 ps, and the trajectory was analysed using the GIST implementation in CPPTRAJ [26,37,38]. Hydration sites were determined using placevent.py and defined to have a radius of 1.5 Å [39]. 

## 4. Conclusions

In conclusion, our binding investigations demonstrate that the AF9 YEATS domain displays selectivity for binding of histones bearing crotonyllysine over the isomeric methacryllysine, two biomedically important histone PTMs. AF9 YEATS was observed to bind histones possessing Kmea with a weaker binding affinity (5–6 fold) than it binds Kcr. MD simulations on AF9 YEATS in complex with H3K18mea and H3K18cr supported these experimental findings by illustrating a more rigid binding conformation of H3K18cr, thus indicating the formation of stable interactions with the protein. Moreover, MD simulations also revealed that the crotonyllysine/methacryllysine-mediated desolvation of the AF9 YEATS domain provides an important contribution to the recognition of both epigenetic marks and the preference of AF9 YEATS for binding Kcr over the regioisomeric Kmea. These findings provide a key molecular insight into binding specificity and pave the way for exploring the functional role of the newly identified methacryllysine in eukaryotic chromatin. The results also provide important molecular knowledge for the rational design and development of AF9 YEATS inhibitors as anticancer drugs. 

## Figures and Tables

**Table 1:** Binding affinities for the recognition of histone H3 peptides by the AF9 YEATS domain. Carried out in triplicates (*n* = 3), and errors are reported as standard error (SE).

Peptide	K_d_ (μM)	N (Stoichiometry)
H3K18mea	114 ± 17	0.99 ± 0.03
H3K18cr	21 ± 2	0.98 ± 0.03
H3K9mea	59 ± 0.3	1.02 ± 0.01
H3K9cr	11 ± 0.8	0.98 ± 0.01

## Data Availability

The data presented in this study are available in the Supplementary Materials of this article.

## Supplementary Materials

The following supporting information can be downloaded at: https://www.mdpi.com/article/10.3390/ijms24087002/s1.
