# Supplementary material for: Molecular Recognition of Methacryllysine and Crotonyllysine by the AF9 YEATS Domain
Source: Int J Mol Sci. 2023 Apr 10;24(8):7002. doi: 10.3390/ijms24087002 (PMC10138300; doi:10.3390/ijms24087002)
Supplement: Supplementary file 1 [file ijms-24-07002-s001.zip › ijms-2339036-supplementary.pdf]

Table of Contents

1. Circular dichroism (CD) spectroscopy

2. Peptide synthesis scheme

3. MALDI-TOF MS spectra and AHPLC chromatograms of histone peptides

4. AF9 expression supporting figures

5. ITC supporting figures

6. MD simulations supporting figures

7. References

1. Circular dichroism (CD) spectroscopy

The thermal denaturation measurement of the protein sample was done using a J-815 CD spectrometer (Jasco), in CD buffer: 25 mM sodium phosphate, 500 mM sodium fluoride, pH 7.4. The protein sample of 11 μM concentration was used for the experiment. The data collection was done in a 0.1 cm thick quartz cuvette, at wavelength 208 nm, temperature range 20 °C to 85 °C, temperature ramp of 1 °C, and delay time at 30 second. The mean residue ellipticity (MRE) was calculated using the following equations (Equation 1).[1]

$$\theta = \frac{signal}{10.l.c.r} \qquad \text{(Equation S1)}$$

θ represent the MRE, *signal* is the measured CD data in millidegree, *l* is the path length, *c* is the concentration of the protein sample in mM, and *r* is the total number of the amino acids in the protein sequence.

The fraction folded at 208 nm (α) was calculated using following equation[2] (Equation 2).

$$\alpha = \frac{(\theta - \theta_D)}{(\theta_F - \theta_D)} \qquad \text{(Equation S2)}$$

θ represent the calculated mean residue ellipticity (MRE), θ_F is the MRE of fully folded protein, and θ_D is MRE of fully denatured protein. A thermal melting curve was then created for the measured data (Fig. S12B).

2. Peptide synthesis scheme

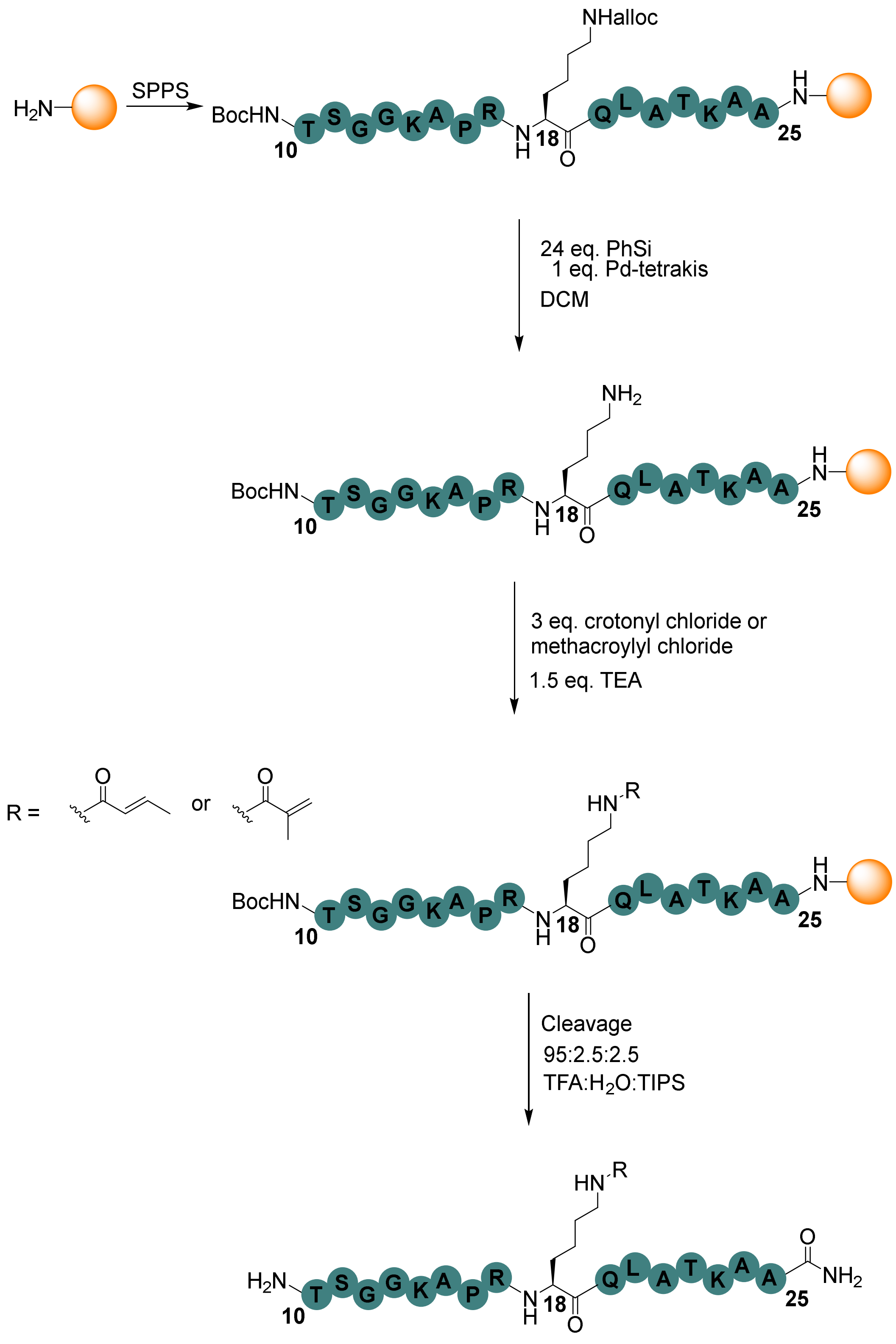

Figure S1. On-resin synthesis of crotonyllysine- and methacryllysine-modified histone peptides.

3. MALDI-TOF MS spectra and AHPLC chromatograms of histone peptides

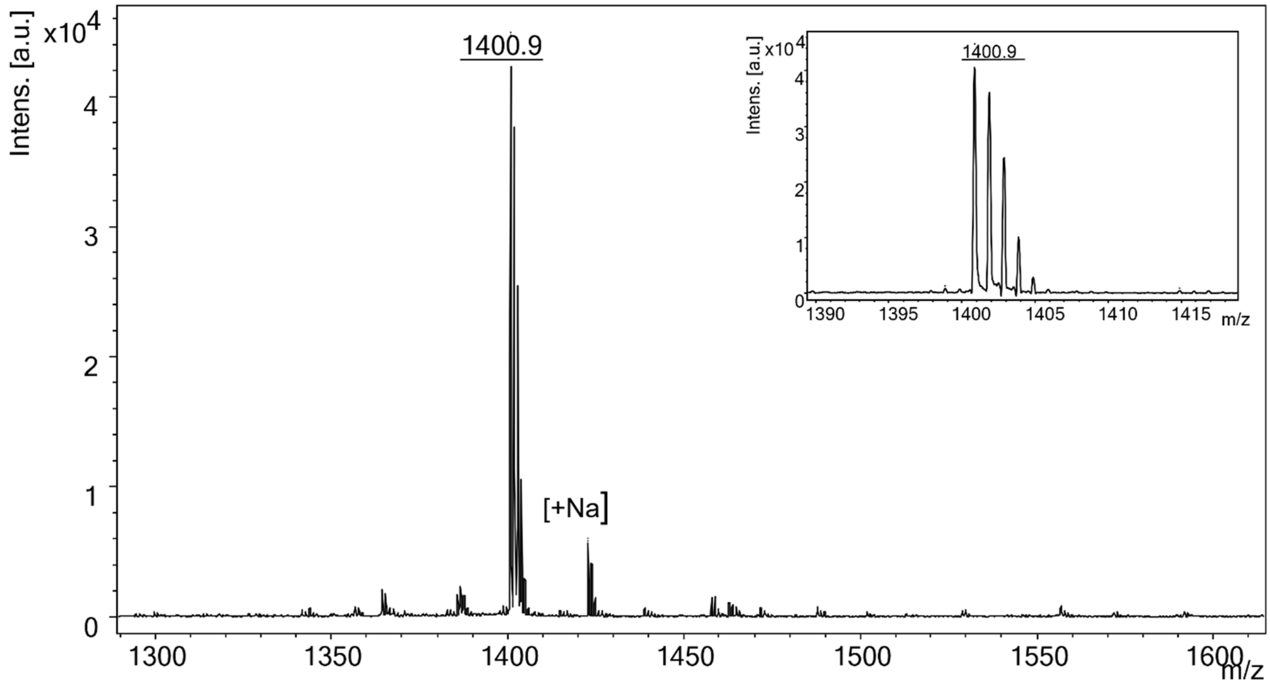

Figure S2. MALDI-TOF MS data for the H3K9cr peptide (*m/z* found 1400.9).

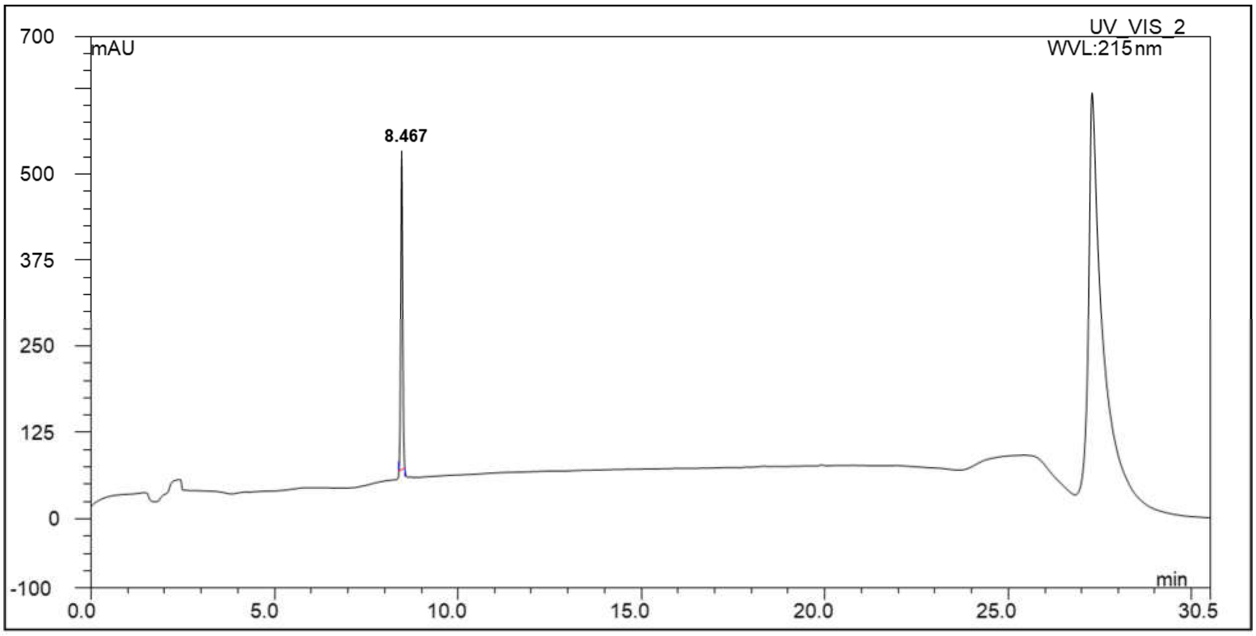

Figure S3. Analytical HPLC of the H3K9cr peptide after RP-HPLC purification. The peptide elutes at 8.5 min.

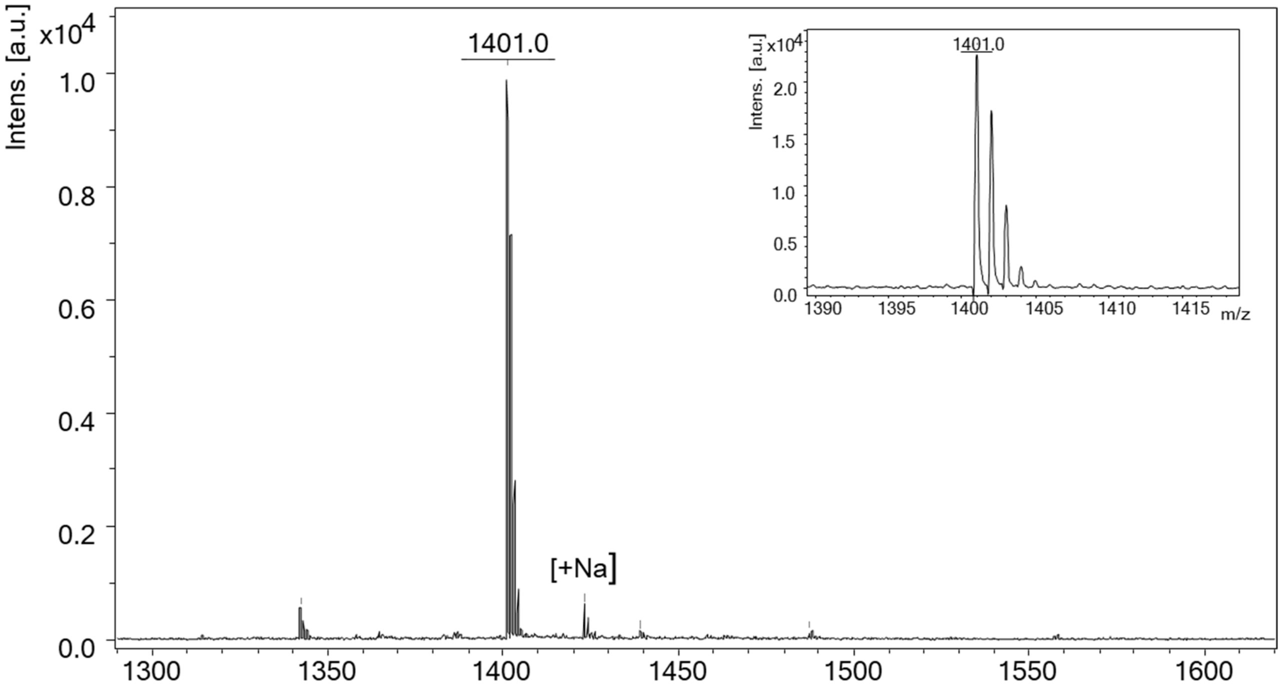

Figure S4. MALDI-TOF MS data for the H3K9mea peptide (*m/z* found 1401.0).

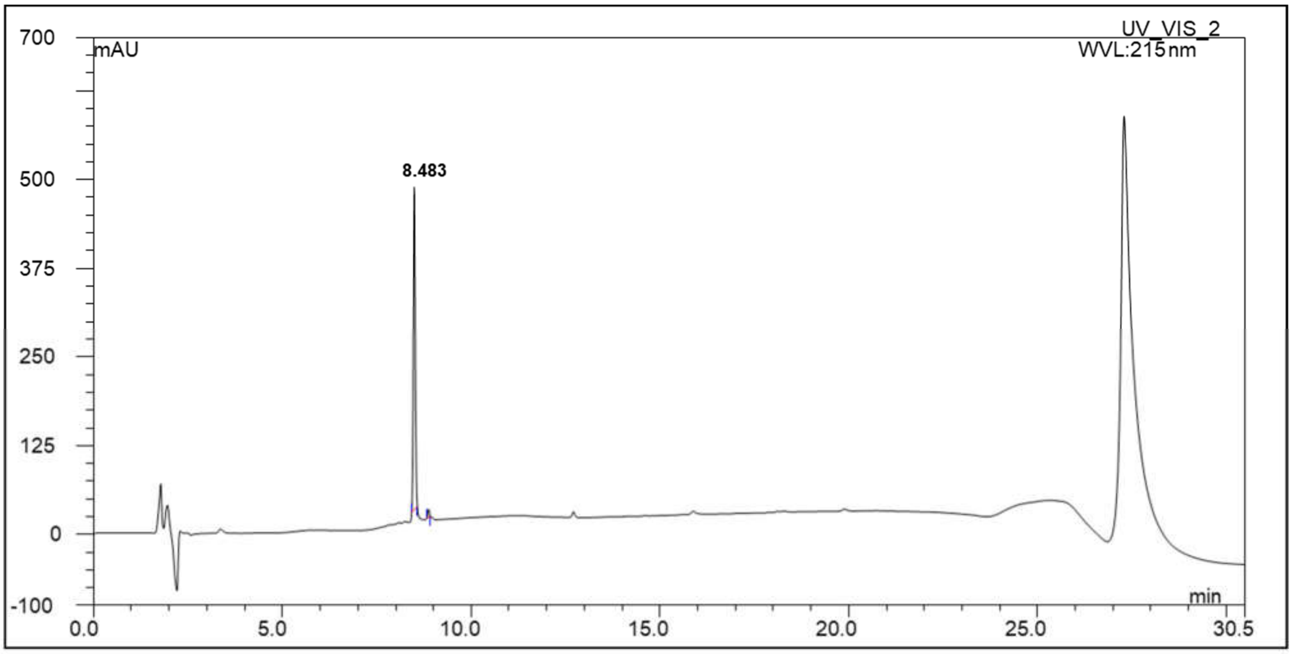

Figure S5. Analytical HPLC of the H3K9mea peptide after RP-HPLC purification. The peptide elutes at 8.5 min.

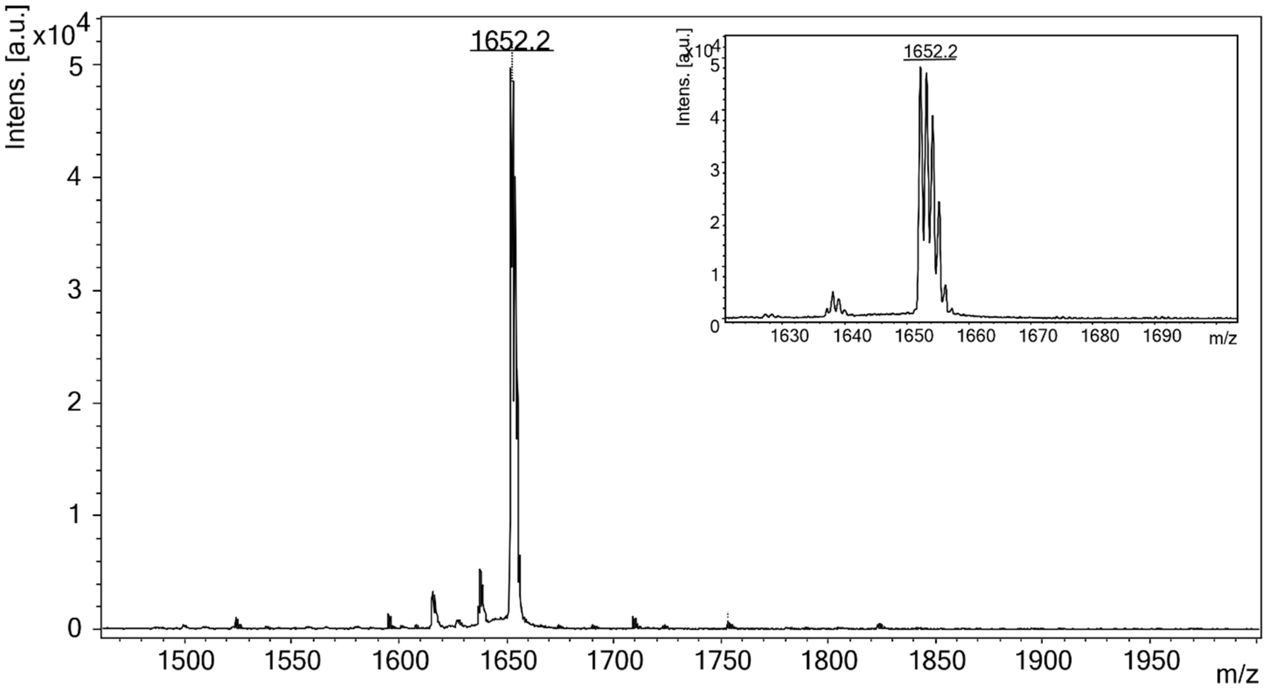

Figure S6. MALDI-TOF MS data for the H3K18cr peptide (*m/z* found 1652.2).

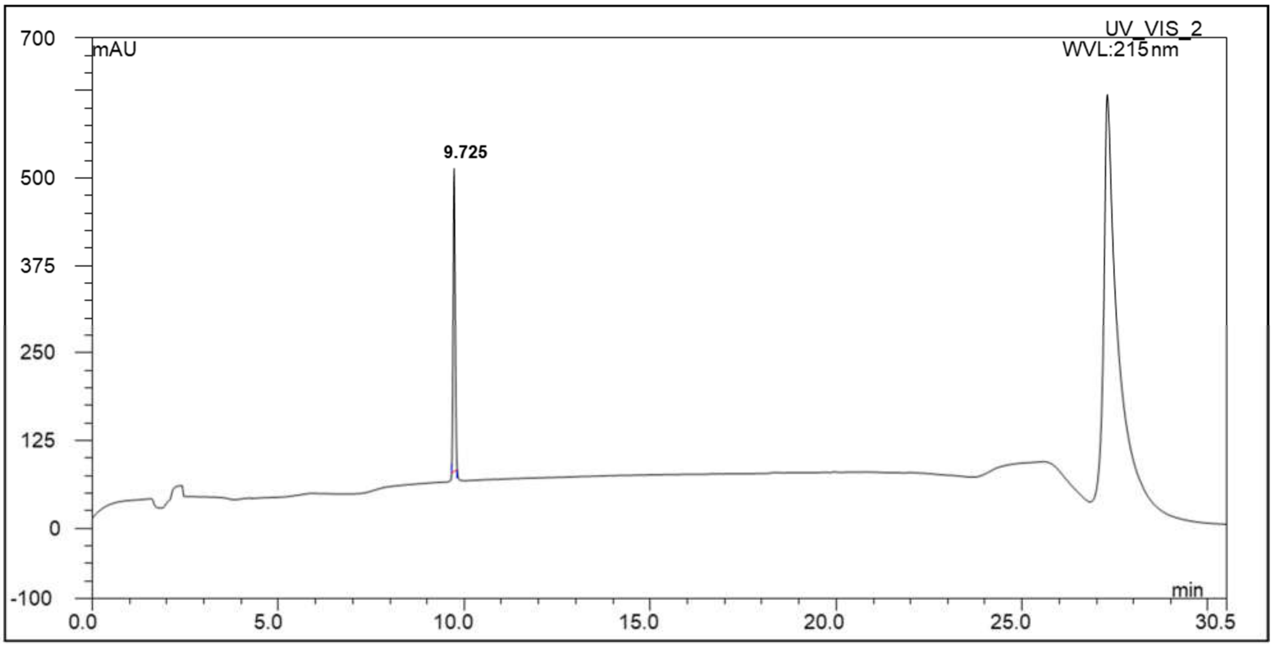

Figure S7. Analytical HPLC of the H3K18cr peptide after RP-HPLC purification. The peptide elutes at 9.7 min.

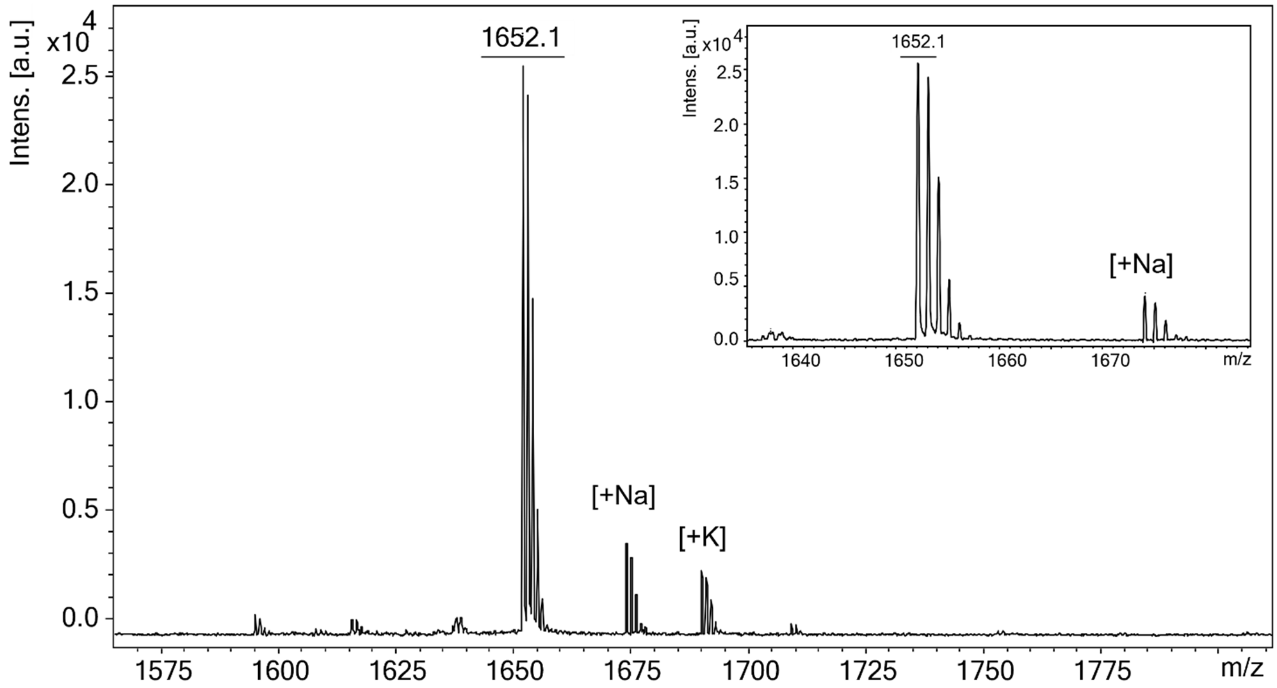

Figure S8. MALDI-TOF MS data for the H3K18mea peptide (*m/*z found 1652.1).

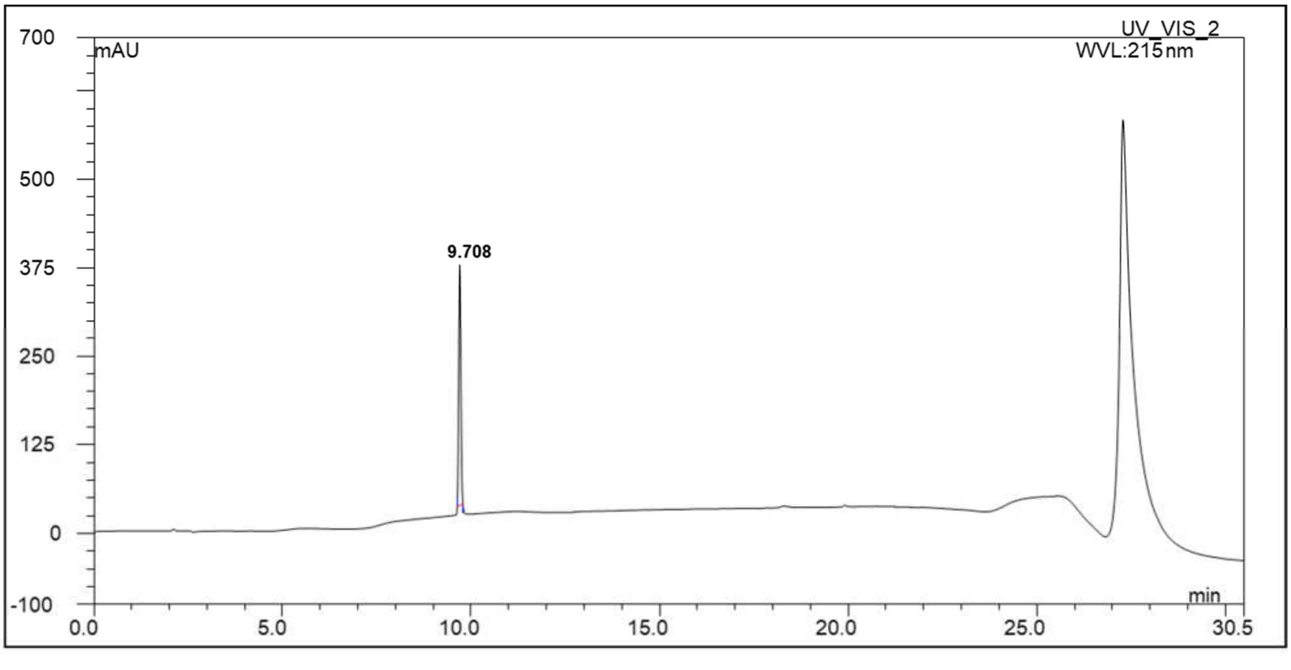

Figure S9. Analytical HPLC of the H3K18mea peptide after RP-HPLC purification. The peptide elutes at 9.7 min.

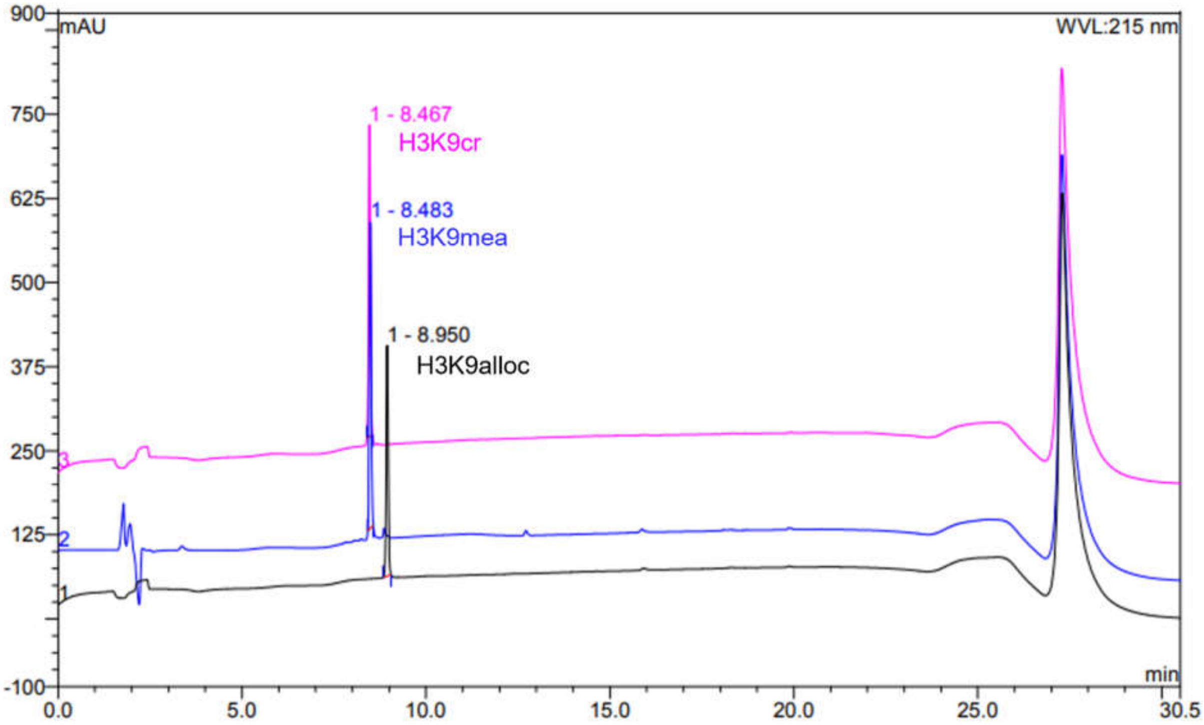

Figure S10. Overlaid analytical HPLC of H3K9cr (pink), H3K9mea (blue) and H3K9alloc (black). Peptides elute at 8.5 min, 8.5 min and 9.0 min, respectively.

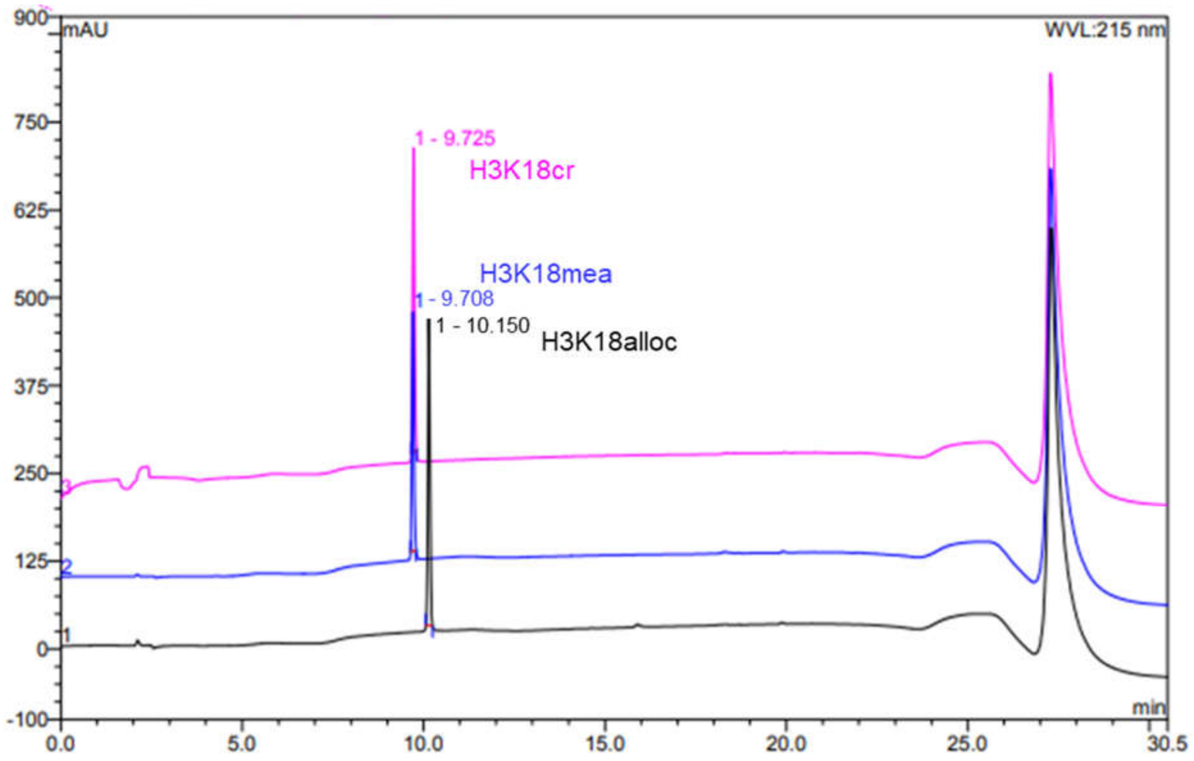

Figure S11. Overlaid analytical HPLC of H3K18cr (pink), H3K18mea (blue) and H3K18alloc (black). Peptides elute at 9.7 min, 9.7 min and 10.2 min, respectively.

Table S1. Overview of synthesized histone peptides and respective m/z values.

Peptide	Sequence	m/z calculated	m/z found
H3K9cr	TKQTARKcrSTGGKA	1400.8	1400.9
H3K9mea	TKQTARKmeaSTGGKA	1400.8	1401.0
H3K18cr	STGGKAPRKcrQLATKAA	1651.9	1652.2
H3K18mea	STGGKAPRKmeaQLATKAA	1651.9	1652.1

4. AF9 expression supporting figures

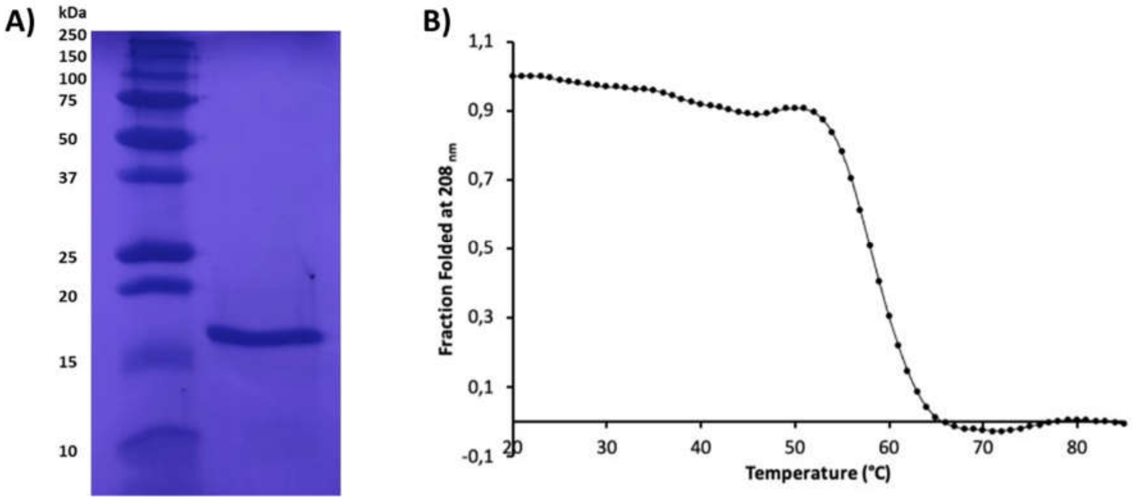

Figure S12. Expression and purification of the AF9 YEATS domain. A) SDS-PAGE image of AF9 YEATS and B) Measured Tm curve of AF9 YEATS using CD spectroscopy.

5. ITC supporting figures

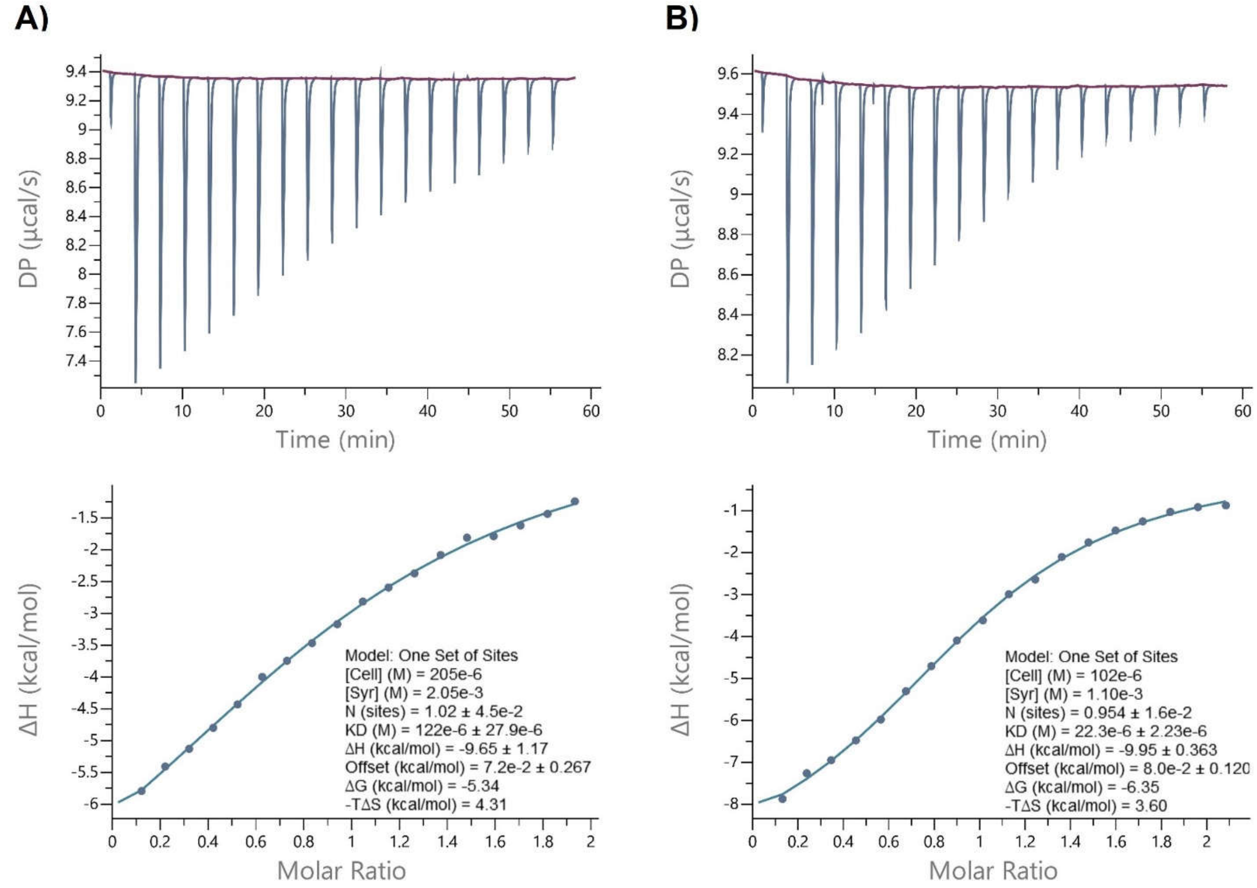

Figure S13. ITC curves showing difference in binding affinity of H3K18mea and H3K18cr to AF9 YEATS. A) Experimental ITC raw and fitting curves of representative binding of H3K18mea to AF9 YEATS, and B) Experimental ITC raw and fitting curves of representative binding of H3K18cr to AF9 YEATS.

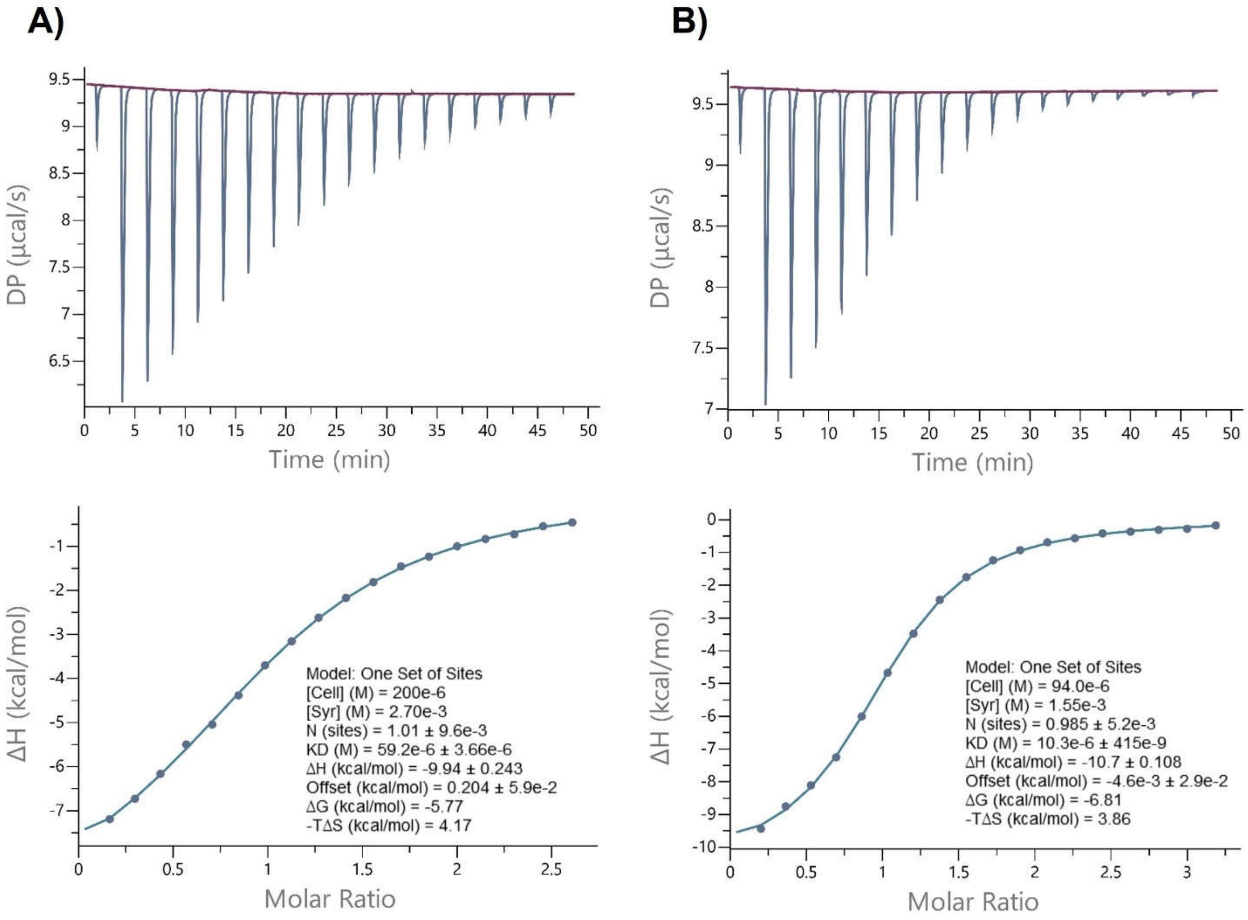

Figure S14. ITC curves showing difference in binding affinity of H3K9mea and H3K9cr to AF9 YEATS. A) Experimental ITC raw and fitting curves of representative binding of H3K9mea to AF9 YEATS, and B) Experimental ITC raw and fitting curves of representative binding of H3K9cr to AF9 YEATS.

6. MD simulations supporting figures

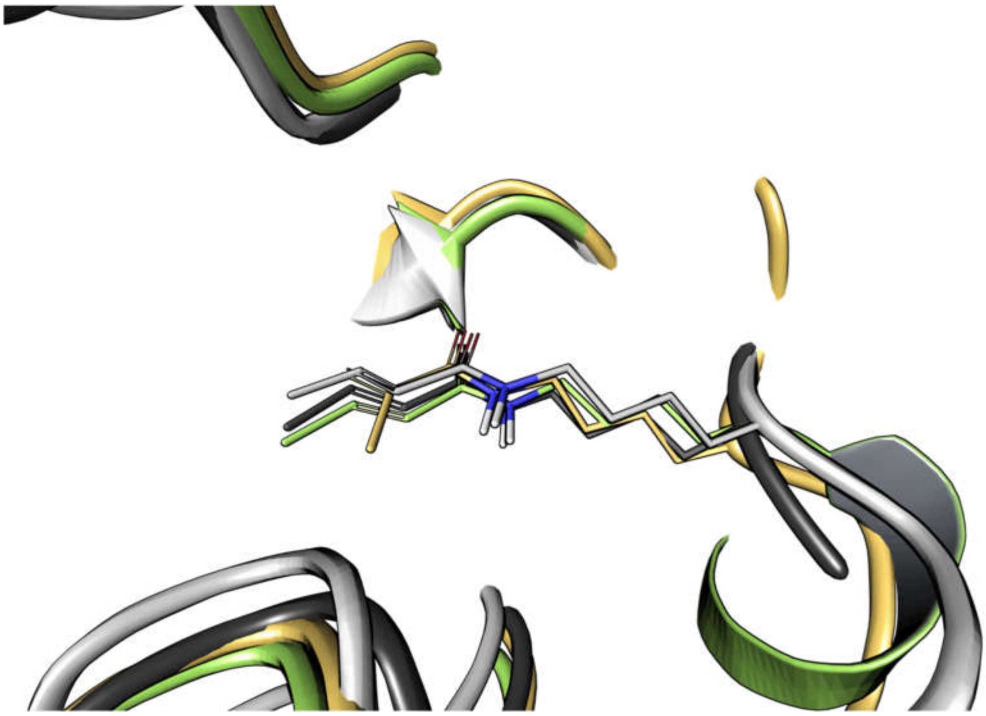

Figure S15. Comparison of the binding conformation of H3K18cr (light gray, PDB ID: 2NDG), H3K9cr (dark gray, PDB ID: 5HJB), the average conformation of H3K18cr from the last 250 ns of the MD simulation (green) and the average conformation of H3K18mea from the last 250 ns of the MD simulation (orange).

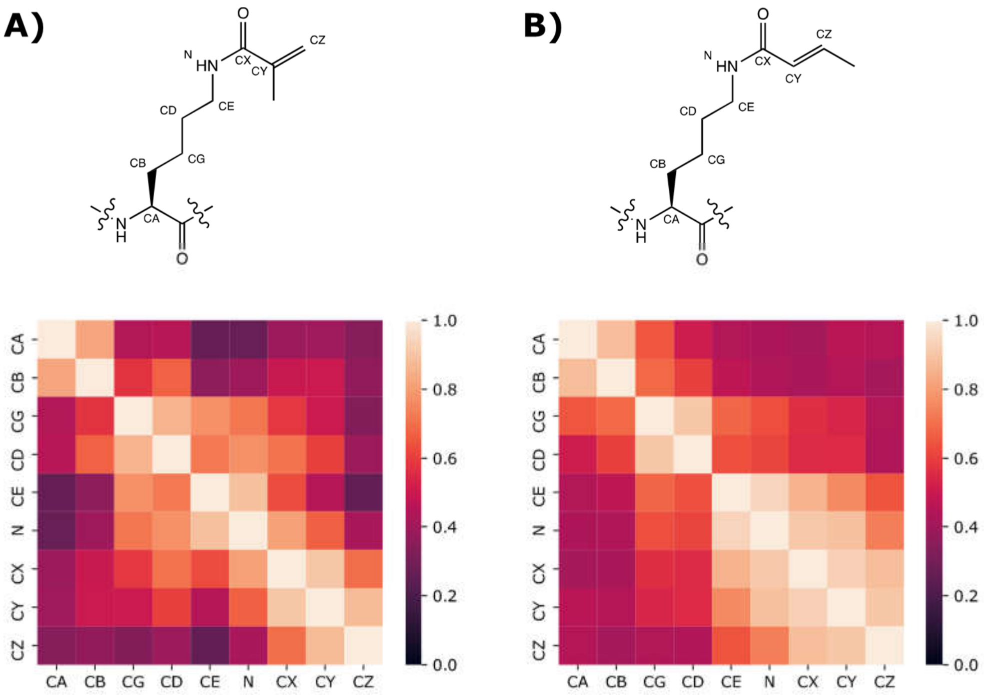

Figure S16. Correlation coefficient matrices of the positions of the atoms in A) the H3K18mea side chain, and B) the H3K18cr side chain.

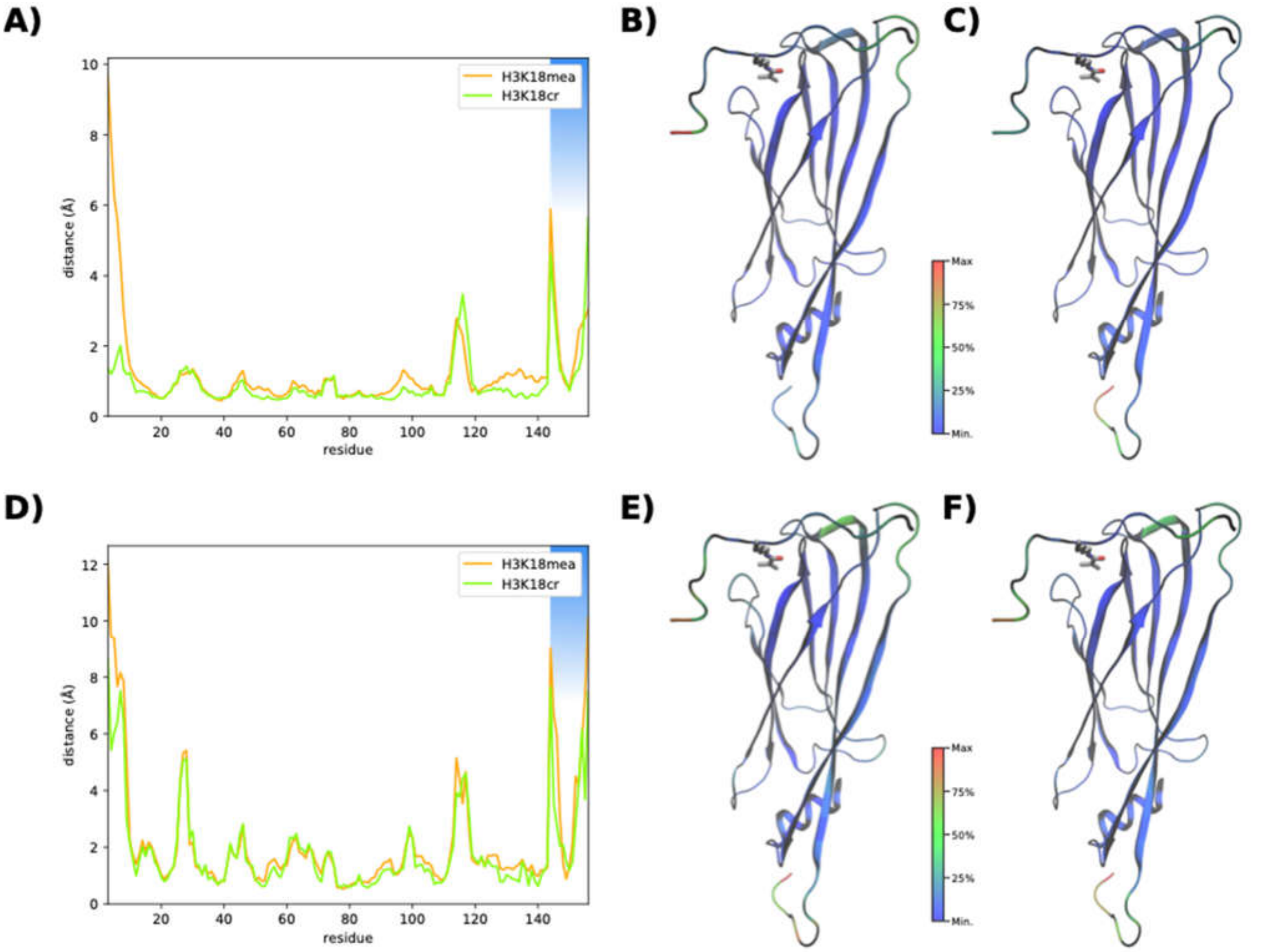

Figure S17. A) Plot of the protein's root mean squared fluctuations (RMSF) from the MD simulations. B,C) Structure of AF9 YEATS with the secondary structure coloured according to the RMSF of H3K18cr and H3K18mea simulations, respectively. D) Plot of root mean squared deviations (RMSD) of the protein from the MD simulations compared to the starting structure. E,F) Structure of AF9 with the secondary structure coloured according to the RMSD of the H3K18cr and H3K18mea simulations, respectively. Blue shaded area: H3K18 peptide.

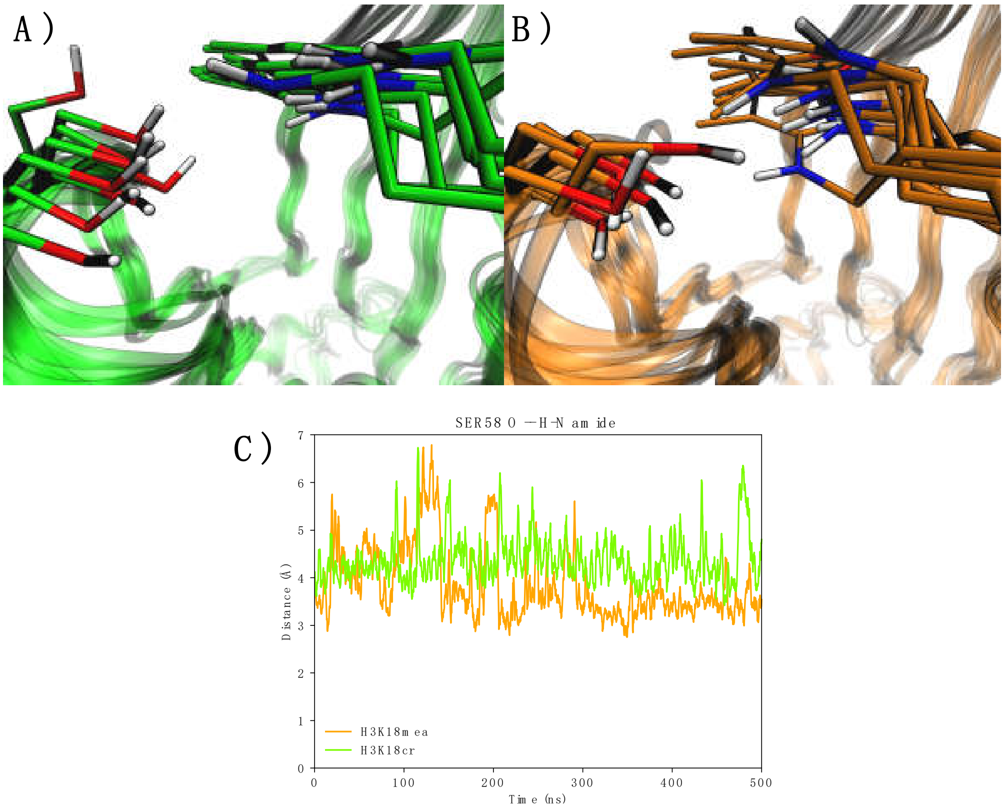

Figure S18. The conformation of Ser58 during the MD simulations. A,B) snapshots from the simulation of (A) H3K18cr, and (B) H3K18mea. C) The distance between the amide H of the bound residue and the oxygen of Ser58.

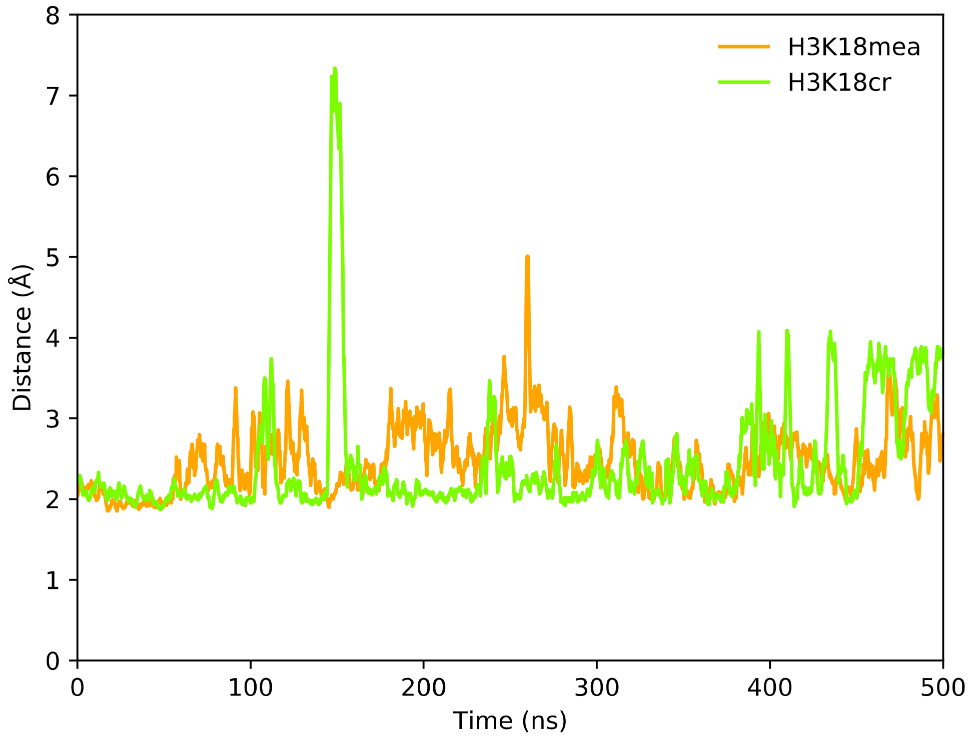

Figure S19. O--H distance of the hydrogen bonds between acyllysine (CO) and Tyr78 (NH) backbone displayed in Fig. 3 during the MD simulations of H3K18mea and H3K18cr.

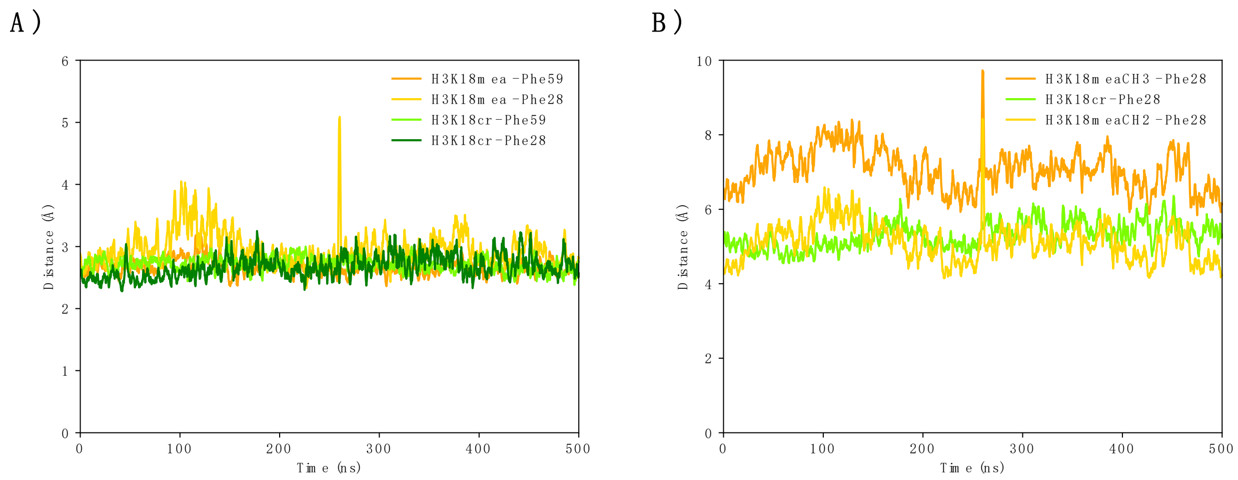

Figure S20. A) Minimum distance between AF9 YEATS binding site phenylalanines and the bound residues during the MD simulations. B) Distance between the center of the Phe28 aromatic ring and the carbon of the methyl/methylidene group of Kcr/Kmea.

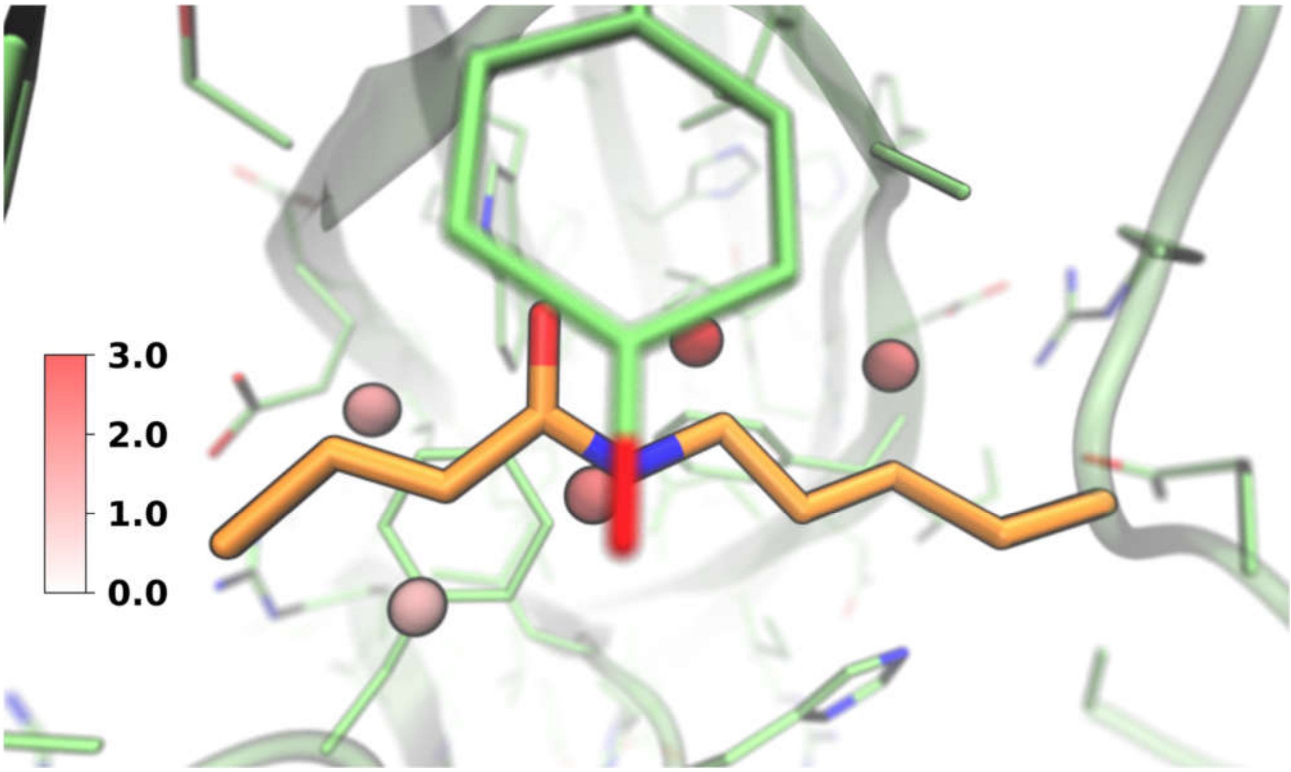

Figure S21. Hydration sites at the methacryllysine/crotonyllysine binding site of AF9 YEATS. Energies are in kcal mol^{-1}.

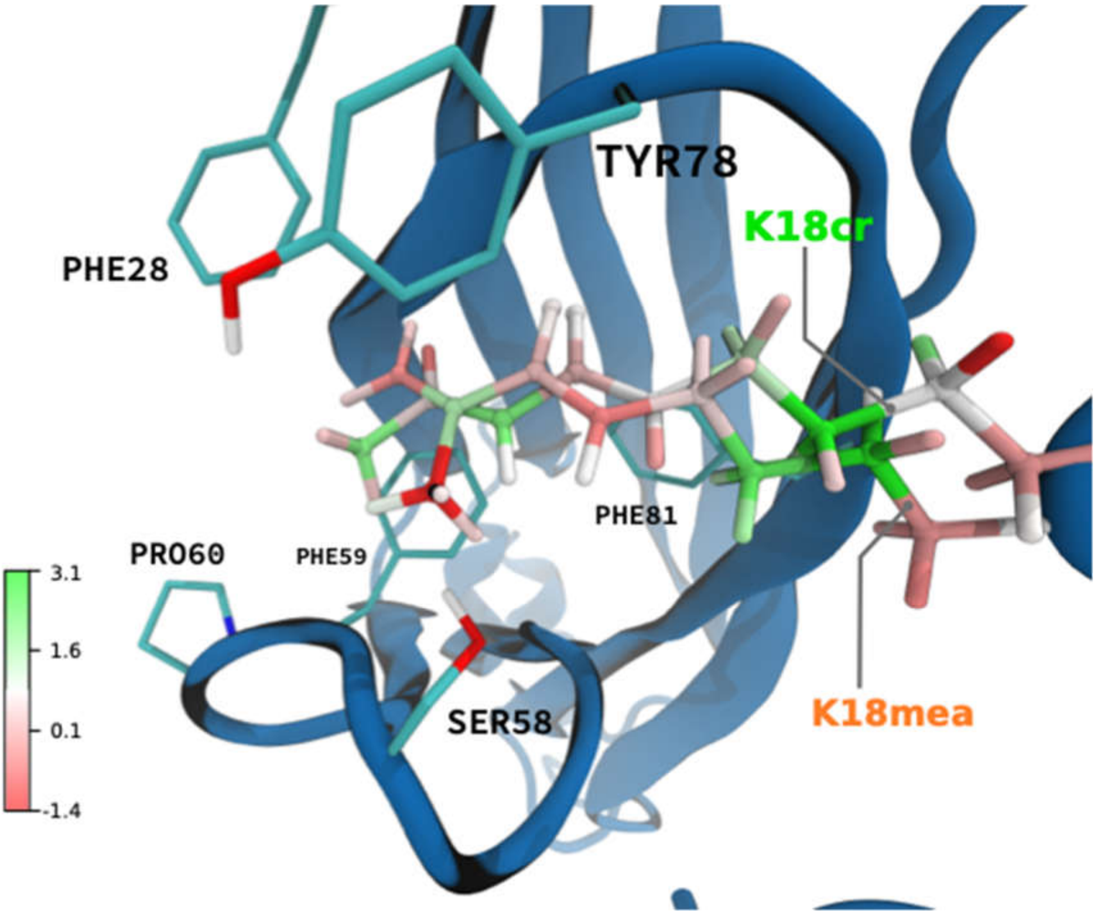

Figure S22. Aligned coordinates of the average structure from the MD simulations of H3K18mea, H3K18cr and the apo AF9 YEATS simulation. The atoms of K18mea and K18cr are color coded according to the solvation energy of the grid points encapsulated by their van der Waals radius. Energies are in kcal mol^{-1}.

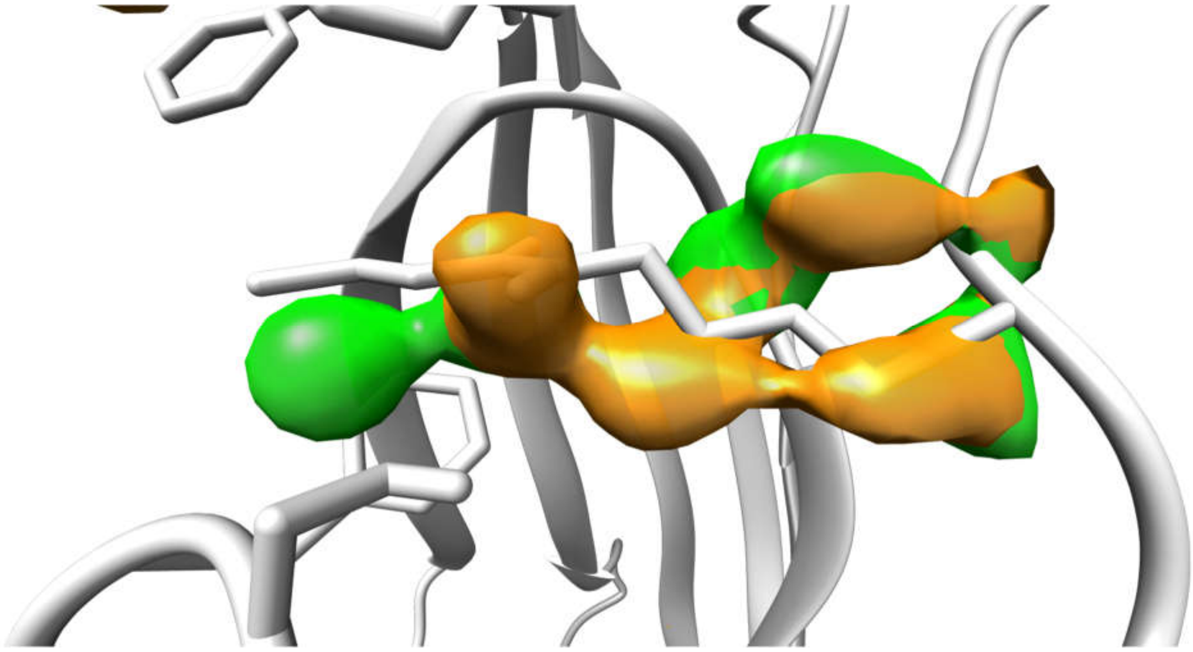

Figure S23. Volumes of the AF9 YEATS binding site, which are desolvated in the simulations of AF9 YEATS bound to H3K18mea (orange) and H3K18cr (green) but are solvated in the apo simulation. A gaussian filter is applied to smoothen the surfaces. Volumes are determined by subtracting densities of water oxygens in the holo MD simulations of AF9 YEATS from the densities in the apo simulations.

7. References

1. Krone M.W.; Travis, C.R.; Lee, G.Y.; Eckvahl, H.J.; Houk, K.N.; Waters, M.L. *J. Am. Chem. Soc.*, **2020**, *142*, 17048-17056.
